# The Role of Regulatory B cells in Kidney Diseases

**DOI:** 10.3389/fimmu.2021.683926

**Published:** 2021-05-24

**Authors:** Wang Long, Hedong Zhang, Wenjia Yuan, Gongbin Lan, Zhi Lin, Longkai Peng, Helong Dai

**Affiliations:** ^1^ Department of Kidney Transplantation, The Second Xiangya Hospital of Central South University, Changsha, China; ^2^ Graduate School of Medical and Dental Science, Department of Pathological Cell Biology, Tokyo Medical and Dental University, Tokyo, Japan; ^3^ Clinical Research Center for Organ Transplantation in Hunan Province, Changsha, China; ^4^ Clinical Immunology Center, Central South University, Changsha, China

**Keywords:** regulatory B cells, Breg, kidney disease, IL-10, transplantation

## Abstract

B cells, commonly regarded as proinflammatory antibody-producing cells, are detrimental to individuals with autoimmune diseases. However, in recent years, several studies have shown that regulatory B (Breg) cells, an immunosuppressive subset of B cells, may exert protective effects against autoimmune diseases by secretion of inhibitory cytokines such as IL-10. In practice, Breg cells are identified by their production of immune-regulatory cytokines, such as IL-10, TGF-β, and IL-35, however, no specific marker or Breg cell-specific transcription factor has been identified. Multiple phenotypes of Breg cells have been found, whose functions vary according to their phenotype. This review summarizes the discovery, phenotypes, development, and function of Breg cells and highlights their potential therapeutic value in kidney diseases.

## Introduction

B lymphocytes play a critical role in adaptive immune system by secreting antibodies. They also present antigens for the activation of T cells and produce several essential cytokines. A small population of B cells known as regulatory B (Breg) cells demonstrates the ability of regulating immune responses. Breg cells are a subtype of B cells that were first discovered in 1974 when two studies described the suppressive function of B cells that could delay hypersensitivity independently (1, 2). However, few studies have focused on suppressive B cells till 1996. Wolf et al. found that in the development of experimental autoimmune encephalomyelitis (EAE), commonly considered as an animal model of multiple sclerosis (MS) mediated by CD4^+^ T cells, EAE is inducible without the exhibition of differences in disease onset or severity between wild-type and B cell-deficient mice. However, B cell-depleted mice recovered with increased difficulty, highlighting the modulation function of B cells in acute EAE (3). Subsequently, an increasing number of studies have found that B cells with regulatory functions are involved in the development of not only EAE but also in other inflammatory diseases (4, 5). To date, these regulatory B cell-induced immune response suppression has been observed in autoimmune diseases, HIV, pregnancy, inflammatory diseases, and transplant immunity, among others. In this review, we have described the different phenotypes and functions of Breg cells and focused on their role in several types of kidney-related diseases, as well as potential challenges in the study of these cells in the future.

## What Is the Phenotype of Breg Cells?

With gradual advances in the existing knowledge since their discovery, Breg cells have not been restricted to a single phenotype, but rather displaying several phenotypes, in both humans and mice.

### Phenotypes of Breg Cells Identified in Humans

B cells that regulate immune responses were initially found to exert their suppressive function in some specific diseases by producing interleukin-10 (IL-10). Subsequently, several studies have shown Breg cells exert suppressive functions mainly by secretion of IL-10 which also is the most important cytokine in Breg cells (6–10). It should also be noted that in certain circumstances IL-10 can serve as an enhancing role in the immune responses of B cells (11, 12) and CD8 T cells (13, 14). The most frequently observed phenotype for IL-10-producing B cells in peripheral blood is transitional CD19^+^CD24^hi^CD38^hi^ B cells (9, 15–20), since IL-10^+^ B cells are abundant in this phenotype. Other phenotypes include plasmablasts (CD19^+^CD27^int^CD38^+^) (21) and regulatory B1 cells (Br1: CD19^+^CD25^+^CD71^+^CD73^-^) (22). Depending on the expression of CD27, IL-10^+^ B cells can be divided into naïve IL-10^+^ B cells (CD19^+^CD27^-^IL-10^+^) and memory IL-10^+^ B cells (CD19^+^CD27^+^IL-10^+^), wherein the ratio of naïve/memory IL-10^+^ B cells may hint at its function (7).

Additionally, Breg cells exhibit other markers. While transcription factor Forkhead Box P3 (Foxp3) is known to be expressed on several immune cells and is commonly regarded as a marker for Tregs, it has also been identified as a transcription factor of Breg cells, namely CD19^+^CD5^+^ B cells; in contrast, CD5^-^ B cells do not express Foxp3 (17, 23–25). Another transcription factor should be noticed is T cell immunoglobulin and mucin domain 1 (Tim-1), a member of Tim family. It is first discovered on the cell surface of T cells and dendritic cells (DCs) and plays a crucial role in immune response regulation (26–28). More recently, B cells have also been found to express Tim-1 (29–31). Human IL-10^+^ B cells also express Tim-1, Tim-1^+^IL-10^+^ B cells are reported to suppress certain autoimmune diseases in human (32). Moreover, Tim-1 may prove to be a better marker for the identification of IL-10-producing B cells than CD5^+^CD1d^+^, since Tim-1 is predominantly expressed on IL-10^+^ B cells in humans (32). While not all of the Breg cells play a protective role in diseases, tumor-evoked Breg cells (tBreg cells), which are functionally different from conventional Breg cells, play negative roles in the occurrence of lung metastasis. The phenotype of tBreg cells is CD19^+^CD25^+^CD81^hi^ (33). tBreg cells reportedly dampen the immune system in breast cancer and the mechanism relies not on the secretion of IL-10, IL-35, or activation of other suppressive pathways, but on the secretion of transforming growth factor-*β* (TGF-*β*) to facilitate Foxp3^+^ Treg cells (34).

### Phenotypes of Breg Cells in Mice

The phenotypes of Breg cells vary considerably between humans and mice. In a murine study, Breg cells were mainly defined as IL-10-producing B cells, which varied from the IL-10^+^ B cell population in the peripheral blood and spleen. Interestingly, according to the analysis of tiger mice, a type of IL-10 reporter mouse, the majority of IL-10-producing cells in the spleen of WT mice comprise B cells, and not T cells (6). The majority of IL-10-producing T cells comprise CD4^+^ T cells (TCRβ^+^CD4^+^) (83% ± 2%), and most IL-10^+^ B cells include follicular (FO) B cells (CD19^+^CD23^+^CD21^int^) (41% ± 3%), while other IL-10-producing cells include neutrophils, monocytes/macrophages, and myeloid DCs. IL-10-producing B cells in the spleen include FO B cells (CD19^+^CD23^+^CD21^int^), marginal zone (MZ) B cells (CD19^+^CD23^-^CD21^hi^), transitional T1+T2 B cells (CD19^+^AA4/CD93^+^), plasma/plasmablast cells (CD19^+^/B220^lo/-^CD138^+^), and B1 a/b cells (CD19^+^CD138^-^CD21^-^CD23^-^CD43^+^CD5^+/-^) (6). In murine studies, the commonly observed IL-10^+^ B cells in the spleen are CD19^+^/B220^lo/-^CD1d^+^CD5^+^ cells (B10 cells) (35–40), since IL-10^+^ B cells are enriched in B10 cells.

Except for B10 cells, other markers or cytokines related to B cells may also play a regulatory role in immune responses. IL-35 secreted by B cells can inhibit inflammation in certain diseases, including inflammatory bowel disease, and such cells are referred to as IL-35-producing B cells (41–43). IL-35-treated mice showed increased abundance of CD4^+^CD25^+^Foxp3^+^ Tregs and IL-10^+^ Breg cells, indicating that IL-35 secreted by Breg cells might exert positive feedback on Breg cells (44, 45). CD9 is a cell surface glycoprotein that is encoded by a gene belonging to the tetraspanin family and is considered a key marker expressed on the cell surface of IL-10-producing B cells. Furthermore, most IL-10^+^ B cells (87.5% ± 1.69%) express CD9 on their cell surface. CD9^+^ B cells have been found to exert a stronger suppressive function than CD9^-^ B cells, while a small proportion of CD9^-^ B cells are known to also secrete IL-10. When CD9 expressed on B cells is subjected to blockade by anti-CD9 antibody, the suppression of T cell proliferation is inhibited, which is related to the ratio of T/B cells in a co-culture system, indicating that the suppressive function of CD9 is related to the establishment of T-B cell crosstalk in an IL-10-dependent manner (38). Tim-1 is not only expressed on human IL-10^+^ B cells, but also a marker for mouse IL-10^+^ B cells. Interestingly, the expression of Tim-1 in B cells is higher than that in T cells. B cells express Tim-1, including transitional B cells, MZ B cells, FO B cells, and B10 cells, and most IL-10^+^ B cells express Tim-1 on their cell surface. IL-4 and IL-10 secretion from Breg cells can be induced with the use of anti-Tim-1 antibody, demonstrating that Tim-1 ligation can induce B10 cell expansion (**Table 1**) (37).

**Table 1:** Subsets of Breg cells.

Subsets	Mouse	Human	Reference
Transitional 2 marginal zone precursor B cells (T2-MZP cells)	CD19^+^CD21^hi^ CD23^+^CD24^hi^ IgM^hi^IgD^+^	CD23^+^sIgM^hi^ sIgD^+^CD35^hi^	(46–49)
Marginal zone B cells	CD19^+^CD23^-^CD21^+^	–	(50–52)
B10 cells	CD5^+^CD1d^hi^	CD5^+^CD1d^hi^/ CD24^hi^CD27^+^	(51, 53–57)
CD1d^hi^ B cells	CD19^+^CD1d^hi^	CD19^+^CD1d^hi^	(58, 59)
Plasma cells/plasmablasts	CD138^+^CD22^-^	CD27^int^CD38^+^	(21, 60, 61)
Peritoneal B1a B cells	CD19^+^CD5^+^ CD11b^+^	–	(50, 62)
Tim-1^+^ B cells	CD19^+^Tim-1^+^	CD19^+^Tim-1^+^	(37, 63–66)
Immature B cells	–	CD19^+^CD24^hi^ CD38^hi^	(67–73)
Circulating B cells	–	CD19^+^CD25^hi^ CD27^hi^CD1d^hi^CD86^hi^	(74)

## Development and Function of Breg Cells

Breg cells induction *in vitro* under different stimuli may associate to Breg cells development *in vivo*. Breg cells constitute several phenotypes of B cells, including transitional B cells, FO B cells, plasmablasts and so forth. They are enriched in the spleen and peritoneal cavity, while few Breg cells exist in the blood, peripheral lymph nodes, mesenteric lymph nodes, and Peyer’s patches (53). *In vitro*, Breg cells can be expanded when stimulated with LPS (75), thereby inducing IL-10^+^ B cell differentiation into plasma/plasmablast cells after transient IL-10 production. After subjection to LPS stimulation for a period of 3 days, B10 cells gradually express CD43 and GL7 activation markers for antibody-secreting cells. Furthermore, the proportion of plasma/plasmablasts in IL-10^+^ B cells increased with an increase in the expression of the plasma/plasmablast-associated transcription factors, namely *blimp1*, *xbp1*, and *irf4*, which are related to plasma/plasmablast cell expansion (76). IL-10^+^ B cells can also be induced by anti-CD40 antibody (75, 77), anti-Tim-1 antibody (37), PMA/ionomycin (78), IL-21 (77, 79) and IL-35 (43, 44, 80). For example, IL-10^+^ B cells that mature into functional IL-10^+^ B cells rely on IL-21 and CD40 signaling activation, whereas IL-10 is not necessary for IL-10^+^ B cell development (75, 77, 79), IL-21 secreted from follicular helper T cells (Tfh) mediates the expansion of B10 cells *via* the phosphorylation of STAT3 (39, 40). Interestingly, IL-21-induced Breg cells exhibit a phenotype that expresses granzyme B, which is a serine protease that is expressed in the granules of NK cells and cytotoxic T cells. Granzyme B secreted by Breg cells inhibits T cell proliferation and the degradation of TCR *ζ*-chain (81, 82). Furthermore, IL-35 is also involved in the development and function of Breg cells. Breg cell-secreted IL-35 is a cytokine that exerts an inhibitory on the immune response. IL-35 induces the production of Treg cells, IL-10-producing Breg cells, and IL-35-producing Breg cells, indicating that Breg cell development is dependent on IL-35 signaling (43, 44, 80).

Breg cells regulate immune responses by many ways, such as anti-inflammatory cytokines IL-10, IL-35, IL-12 (83), or TGF-*β* (84, 85) secretion, or *via* the Fas-FasL (86) and PD-1/PD-L1 (86, 87) pathways. Through all these pathways, Breg cells regulate the immune system by controlling immune cell differentiation and proliferation. As the major functional cytokine secreted by Breg cells, IL-10 production is related to the B-cell linker protein (BLNK) expression, which is involved in the regulation of the immune response in allergic and autoimmune diseases (88), it regulates the differentiation of Th1 cells and Th17 cells and inhibits T cell cytokine secretion resultantly (47). IL-10^+^ B cells also modulate the function of Foxp3^+^ Tregs and CD8^+^ T cells. For example, the levels of IL-10 in the serum are elevated in patients with chronic hepatitis B virus (HBV) infection, and the blockade of IL-10 supports the function of virus-specific CD8^+^ T cells. Both the frequency of IL-10^+^ B cells and the secretion of IL-10 from IL-10^+^ B cells are facilitated in these patients, suggesting that Breg cells inhibit CD8^+^ T cell function in an IL-10-dependent manner (9). Additionally, IL-10^+^ B cells promote CD4^+^Foxp3^+^ T cell proliferation both *in vivo* and *in vitro* (89, 90) (91, 92). Evidence also indicates that IL-10 produced by Breg cells induces CD4^+^ T cell apoptosis (93). IL-10^+^ B cells function is also different among naïve IL-10^+^ B cells and memory IL-10^+^ B cells. Naïve IL-10^+^ B cells are involved in the prevention of immune responses in autoimmune diseases, while memory IL-10^+^ B cells prevent disease exacerbation (94). The ratio of naïve/memory IL-10^+^ B cells may be an indicator of the major function of IL-10^+^ B cells in specific diseases (7). Breg cells also regulate IgG production, with B10 cell depletion enhancing IgG production in WT mice (35). In a human study, Breg cells were found to impair the function of IgG4-producing B cells (22). Moreover, Breg cells inhibit the differentiation of CD4^+^ T cells into Tfh cells and suppress the antibody production mediated by Tfh cells (91, 92). Breg cells not only participate in the suppression of peripheral immune response but also perform inhibitory functions in the brain. They are capable of inhibiting inflammation and central nervous system damage resulting from infiltrating pro-inflammatory cells (95). Collectively, even though only 1%–2% of splenic B cells are IL-10^+^ B cells, they play a critical role in the regulation of immune responses (53) by suppressing immune responses and by ameliorating autoimmune diseases.

Regarding tBreg cells, which play a negative role in immune responses, anti-CD20 antibody treatment facilitates tumor escape from the immune system *via* the enrichment of tBreg cells that express low levels of CD20. Thus, using anti-CD20 antibody may enrich tBreg cells, which impairs the immune system and promotes breast cancer development (33).

However, the role of Breg cells in mice and human are somehow not always the same. It is also reported that CD19^+^CD24^hi^CD38^hi^ Breg cells are enriched in some SLE patients, together with elevated serum IL-10 level from Breg cells and reduced IL-10R in circulating lymphocytes, demonstrating that IL-10 secreted from Breg cells in human is not necessarily protective in autoimmune diseases, and can be targeted in some cases (96).

## What Is the Role of Breg Cells in Kidney Disease?

Breg cells exert protective effects in systemic diseases that affect the kidney, including allograft rejection (46, 97), systemic lupus erythematosus (SLE) (98), type 1 diabetes (T1D) (99, 100), anti-neutrophil cytoplasmic antibody-associated vasculitis (AAV) (101), Sjogren’s syndrome (SS) (102), Immunoglobin G4-related disease (IgG4-RD) (103), and IgA vasculitis with nephritis (IgAVN) (104), among others. Breg cell populations tend to be reduced in the above-mentioned kidney-targeting diseases. The transfer or increase of Breg cell numbers and the increased secretion of inhibitory cytokines from Breg cells alleviates disease, leading to improved kidney function. Several diseases, including AAV, T1D, and SLE, result from the generation of autoantibodies and inflammatory cytokine secretion, with or without T cell infiltration. An increased number of Breg cells reduces antibody production and CD4^+^ and CD8^+^ T cell infiltration, and promotes the infiltration of Treg cells, thereby leading to disease remission.

### Kidney Transplantation

B cells play an important role in graft rejection by producing donor-specific antibodies, while increasing evidence support the role of B cells in the induction of tolerance. Long-term acceptance was more likely to be induced in kidney-transplanted rats administered with donor-derived B cells compared to donor-derived T cells (105). The importance of B cells in tolerance induction has also been demonstrated in human studies (67, 106). Based on the studies conducted using B cells collected from operational tolerant (OT) recipients, B cells were found to produce higher levels of IL-10 (107). Furthermore, the frequency of naïve B cells, memory B cells, and Breg cells increased and tended to be normal (15, 108). Breg cells may inhibit T cell function *via* the direct interaction of T:B cells (109). Different from most settings, IL-10 shows counter-regulatory effects in the setting of anti-CD45RB-induced tolerance. In anti-CD45RB-induced heart allograft mice model, IL-10 deficiency or IL-10 neutralization was found to improve chronic allograft vasculopathy and reduce allograft reactive antibody production. However the underlying mechanism behind this abnormality is not yet known in this specific setting (110). Breg cells in patients with tolerance were found to be similar to those in healthy individuals, while patients who experienced chronic rejection showed impaired Breg cell population (15). The immunologic injury targeting allografts is markedly related to the IL-10/TNF-*α* expression ratio on Breg cells (111). Therefore, elevated circulating IL-10^+^ Breg cells could be a marker for relatively lower risk of antibody-mediated rejection (20). More convincing evidence indicates that Breg cells are protective in inducing the tolerance or preventing the rejection of allografts in cases with IL10^-/-^ on B cells or B cell depletion, which are more likely to be rejected; furthermore, evidence also indicates that the adoptive transfer of IL10^+/+^ marginal zone precursor regulatory B cells in recipients prevents rejection (46, 97). The main mechanisms involved in the induction of tolerance by Breg cells are as follows: (1) the regulation of IL-10^+^ Breg cells promote Foxp3^+^ T cell proliferation (90, 112), inhibit the proliferation (113) and induce the apoptosis (93) of CD4^+^ T cells, and dampen the function of CD8^+^ T cells (9); (2) activation of other cytokines and pathways.

### Lupus Nephritis (LN)

Kidney function is commonly investigated in the prognosis of patients with SLE. As autoantibodies are the key factors in the pathogenesis of SLE and LN, increasing evidence shows the significant role of Breg cells in LN (98). Early studies have reported that the number of IL-10-producing B cells is increased in patients with SLE (114, 115). However, in patients with new-onset SLE (116) and LN patients (117), the IL-10^+^ B cell population is decreased. The percentages of CD19^+^CD24^hi^CD38^hi^ subsets, including putative Breg cells in PBMCs, between SLE patients and healthy controls are similar (68, 117). However, Breg cells in the PBMCs of SLE patients secrete fewer amounts of IL-10 with an impaired CD4^+^ T cell suppressive capacity (118). Consistent with this finding, CD19^+^CD24^hi^CD38^hi^ Breg cells in SLE patients produced fewer amounts of IL-10 with an impaired suppressive capacity. The suppressive effect of Breg cells on CD4^+^ T helper 1 cells is dependent on IL-10, CD80, and CD86, but is not TGF-β-dependent. This suppression impairment of SLE-derived Breg cells may be related to its inability to upregulate STAT3 phosphorylation upon CD40 engagement (68). Early immature B cells can produce a substantial number of self-reactive antibodies, including ssDNA-reactive antibodies (119, 120). Interestingly, IL-10-producing CD27^−^CD38^int^IgD^+^ pre-naïve B cells in SLE patients secrete fewer amounts of IL-10 with enhanced CD80 and CD86 expression, and this occurrence leads to the loss of self-regulation (120).

The reason for the reduced population of IL-10^+^ Breg cells in SLE patients may be related to the regulatory circuit between plasmacytoid dendritic cells (pDCs) and CD24^+^CD38^hi^ Breg cells (69). In healthy individuals, pDCs can promote the differentiation of both Breg cells and plasmablasts by moderating IFN-α secretion and CD40 signaling. On the other hand, IL-10 produced by Breg cells can restrain the release of IFN-α by pDCs. However, in SLE patients, increased IFN-α production by hyperactive pDCs drives immature B cell differentiation with an inclination toward plasmablast generation. As a result, SLE Breg cells with reduced IL-10 production fail to restrain pDC-derived IFN-α, thus creating a vicious circle. This study also found that in SLE patients responding to rituximab, repopulated B cells contained a normal frequency of CD24^+^CD38^hi^ Breg cells, and this mitigated pDC activation and restored the balance (69). Epratuzumab, which targets CD22, can reportedly inhibit B cell-derived pro-inflammatory IL-6 and TNF-α secretion while maintaining the production of IL-10 (121). This represents a potential therapeutic strategy to disrupt the vicious cycle between pDCs and Breg cells.

Another immunosuppressive agent, prednisolone, is also commonly used in SLE patients. However, the percentage of IL-10^+^ B cells in LN patients is negatively correlated with the daily dose of prednisolone (117) which indicates that the currently available immunosuppressive agents can affect both the effector and regulatory aspects of B cells. This might indicate that the current immunosuppressive treatment strategy failed to treat SLE/LN because of its inability to restore the natural immune balance.

Efforts have been engaged to consider and utilize Breg cells as therapeutic targets for treating LN. Human adipose-derived mesenchymal stem cells (MSCs) can trigger the expansion of IL-10-producing Breg cells *in vitro* and *in vivo* in a mouse model. After MSC treatment in the SLE mouse model, both renal histopathology improvement and autoantibody mitigation have been achieved (122). A similar effect has also been reported with the application of MDSCs, which can be subjected to blockade using an inhibitor of iNOS (89).

### Type 1 Diabetes (T1D)

T1D is an autoimmune disease characterized by the destruction of cells of the islets of Langerhans, particularly beta cells. Autoantibodies, such as an anti-insulin antibody, anti-islet cell antibody, and anti-glutamic acid decarboxylase (GAD) antibody, can be detected in most T1D patients, accompanied by lymphocyte infiltration in the pancreas. Several immunosuppressive therapies have been tested, including those involving the use of cyclosporine A (CsA) and anti-CD3 monoclonal antibodies (123). Although CsA is an effective initial therapeutic strategy to confer protection to the pancreas, it is not appropriate for long-term use due to side effects (123). Although anti-CD3 antibody showed long-term effects in NOD mice, a mouse model for T1D, treatment strategies using the antibody did not achieve success in phase III clinical studies (124).

Despite considerable efforts to develop immunotherapies that target Treg or T cells for the treatment of T1D, thus far, there is a lack of effective immunotherapies. Interestingly, accumulating evidence indicates that Breg cells play an important role in the suppression of the pathology of T1D. B cell depletion by anti-CD20 antibody leads to the long-term remission of T1D in NOD mice, possibly due to the removal of autoreactive B cells and the reduction in autoantibody generation, which is critical for the development of disease in NOD mice. Another critical mechanism is the increase in the number of Foxp3^+^ Treg cells, which may be caused by an altered proportion of regenerated B cells (100, 125–127); however, the anti-CD20 antibody is effective only in the early stage of T1D, since pancreas-infiltrating B cells lose the ability to express CD20 (126). A similar result was obtained when B cells were subjected to depletion with the use of anti-CD22 antibody in NOD mice. When conducting the transfer of the regenerated B cells into NOD mice, Treg cells underwent expansion, anti-inflammatory cytokine secretion from Treg cells increased, and infiltrated T cell populations reduced, indicating that these regenerated B cells were regulatory and might exert a protective effect in T1D (128). Additionally, several studies have shown that Breg cells alleviate T1D pathophysiology in an IL-10-dependent manner in both mice and humans (99, 129). In a study using NOD mice of different ages, long-term normoglycemic NOD mice (average age, 30 weeks) exhibited reduced lymphoid infiltration with an increase in pancreas-infiltrating IL-10-producing B cells compared with glycemic NOD mice. To elucidate the role of IL-10^+^ B cells in NOD mice, a reduction in IFN-γ production by CD4^+^ T cells was observed when the cells were co-cultured with IL-10^+^ B cells and DCs. Additionally, milder disease pathology was observed when IL-10^+^ B cells were transferred with diabetogenic CD4^+^ T cells into NOD mice compared to the mice subjected to transfer with IL-10-producing B cells, demonstrating that IL-10^+^ B cells played a protective role in the development of T1D in NOD mice (99, 130). In a study conducted in humans, the proportion of CD5^+^CD1d^+^ B cells was positively correlated with blood C-peptide levels, a test performed to analyze insulin secretion, indicating that B10 cell reduction was related to islet destruction (129). Taken together, Breg cells seem to play a protective role in the development of type 1 diabetes in an IL-10-dependent manner, suggesting that Breg cells may be a potential target for the treatment of type 1 diabetes.

### Anti-Neutrophil Cytoplasmic Antibody (ANCA)-Associated Vasculitis (AAV)

ANCAs are autoantibodies that target neutrophil cytoplasmic antigens, such as proteinase 3 (PR3) and myeloperoxidase (MPO). Over 75% of the patients with AAV present with rapidly progressive glomerulonephritis, which is an important predictor of mortality (131). Studies have shown that CD19^+^CD24^hi^CD38^hi^ cell populations are decreased in active AAV patients, while its suppressive functions and the ability to produce IL-10 are not altered (101, 132). However, during remission in AAV patients, one study found that this subset was subjected to continuous reduction (101) while another study found that the frequency of this subset did not vary from that of healthy controls. The CD19^+^CD24^hi^CD27^+^ subset, supposedly comprising B10 memory Breg cells, is reportedly reduced during an active disease state and restored during remission (132). Human CD5^+^ B cells are considered to produce IL-10 and TGF-*β* (133, 134). This CD5^+^ B cell population decreases in active AAV patients and rebounds after remission, highlighting its potential role as an indicator of disease activity, remission, and relapse (135, 136). Following B cell depletion with rituximab, a lower CD5^+^ percentage in B cells was correlated with a shorter time of relapse (137). However, whether CD5 alone is a practicable putative surrogate marker for Breg cells and whether its status can be considered as an indicator of AAV disease activity warrant investigation. Another study found that CD5^+^ B cells were inversely correlated with disease activity during relapse after treatment with rituximab; however, they were could not be used to predict the time to subsequent relapse (138).

### Sjogren’s Syndrome (SS)

Sjogren’s syndrome is one of the most common autoimmune diseases, characterized by impaired exocrine function due to lymphocyte infiltration (139). The clinically overt implication of the kidney has been reported in approximately 5% of SS patients, with a low incidence of progression to end-stage disease (140, 141). Most cases of renal involvement comprise tubulointerstitial nephritis (TIN). Biopsies have shown the infiltration of T cells, B cells, and plasma cells (142). SS patients have been reported to present with a higher frequency of CD19^+^CD24^hi^CD38^hi^ Breg cells, whose suppression ability is compromised (102, 143). However, IL-10^+^ B cell populations are substantially lower in both primary SS patients and SS mice models, and such a phenomenon is caused by a decreased production of IL-10 by CD19^+^CD24^hi^CD38^hi^ cells. The adoptive transfer of IL-10-producing Breg cells can be used to ameliorate SS progression in a mouse model, thereby revealing the potential therapeutic effect of Breg cells (144). Another study found no significant difference in the percentage of CD5^+^ B cells compared to that observed in healthy controls; however, IL-21 receptor and Granzyme B expression in CD5^+^ B cells in primary SS patients were markedly enhanced, indicating the elicitation of an increased counter-regulatory reaction (145).

### Immunoglobin G4-Related Disease (IgG4-RD)

IgG4-RD is a rare fibroinflammatory disease histologically characterized by lymphoplasmacytic infiltration enriched with IgG4^+^ plasma cells and with the occurrence of storiform fibrosis. Approximately 15% of the IgG4-RD patients exhibit clinical implications in the kidney (146). In type 1 AIP (pancreatic manifestation of IgG4-RD), CD19^+^CD24^hi^CD38^hi^ Breg cell populations were found to be significantly increased, while the CD19^+^CD24^hi^CD27^+^ subset was decreased. IL-10-producing B cells were found to be similar between type 1 AIP and healthy controls (103). However, in another study involving 48 newly diagnosed IgG4-RD patients, CD19^+^CD24^hi^CD38^hi^ Breg cells showed a marked reduction (143). This may be attributed to the heterogeneity of IgG4-RD.

### IgA Vasculitis With Nephritis (IgAVN)

IgA vasculitis, formally known as Henoch-Schönlein purpura, is a type of IgA-mediated small-vessel vasculitis (147). Studies have reported that, compared with healthy controls, all CD19^+^CD24^hi^CD38^hi^, CD19^+^CD5^+^, and CD19^+^IL-10^+^ subsets are decreased in patients with IgAVN (54, 104). Moreover, even the concentration of IL-10 in the serum has been found to be considerably lower. After treatment, mainly with the glucocorticoid prednisolone, an increase was observed among CD5^+^CD1d^+^, CD5^+^CD1d^+^IL-10^+^, and IL-10^+^ B cell subsets, accompanied by an increase in the serum IL-10 concentration (54).

### Breg Cells in Other Kidney Diseases

Numerical changes or functional alterations of Breg cells have also been observed in other immune-related kidney diseases. IgA nephropathy (IgAN) patients tend to have a lower frequency of CD5^+^CD1d^+^CD19^+^ Breg cells with a reduced IL-10 expression. CD5^+^CD1d^+^CD19^+^ Breg cells and Breg-derived IL-10 concentrations are negatively correlated with serum IgA and Gd-IgA1 levels, respectively (148). Similar findings have been reported in diabetic nephropathy (DN) patients in terms of the CD19^+^CD24^hi^CD38^hi^ Breg subtype (149). However, among patients with hepatitis B virus-associated membranous nephropathy (HBV-MN), higher proportions of CD5^+^CD19^+^ and IL-10^+^CD19^+^ B cells and serum IL-10 levels have been reported (150). These differences could be attributed to various distinct Breg cell phenotypes or Breg cell changes that occur depending on the immunological environment. However, whether these numerical or functional changes in Breg cells are a cause or an effect of immune-related kidney diseases should be elucidated in future studies.

In addition carious studies have shown that renal fibrosis, as an inevitable outcome of nearly every kind of chronic kidney diseases, can be driven by TGF- *β* (151). On the other hand, IL-10 was found as a protective factor in animal studies (152–154). Presumably Breg cells might play a role in renal fibrosis through cytokine production or interactions with other immune cells such as macrophage, T cells etc. However, currently few works has been done regarding the effects of Breg cells on renal fibrosis which calls for further study.

## Discussion

Breg cells regulates immune responses through the secretion of several inhibitory cytokines such as IL-10, IL-35 and TGF-*β* or PD1/PDL1, Fas/FasL pathways, thus facilitate Treg cells, impair CD4^+^ T cells, CD8^+^ T cells, DCs, Th1/Th17 and IgG production. These aspects are crucial to achieve successful treatment of autoimmune diseases, inflammation disease regulation, tumor growth prevention, and transplant tolerance induction. Breg cells exhibit several phenotypes, including transitional B cells, MZ B cells, and plasma/plasmablasts, among others. As a result, there is no single specific marker that can be considered for identifying effective Breg cells. Therefore, Breg cells are generally identified *via* their secretion of suppressive cytokines such as IL-10. Since Breg cells secrete several cytokines, including IL-10, IL-35, and TGF-*β*, to regulate immune responses, IL-10^+^ B cells may constitute a considerable population of Breg cells, but cannot represent all Breg cells.

Breg cells exert protective effects against many inflammatory diseases and tolerance induction, While IL10 KO in B cells result in deterioration of inflammatory disease conditions. The transfer of IL-10^+^ B cells ameliorates autoimmune responses and induces tolerance. Although Breg cells possess properties that are beneficial in the immune response, they cannot be easily utilized in clinical trials. Unlike Treg cells, Breg cell development is not well understood due to its variation in phenotypes. Thus, the identification of a surface marker pattern or lineage-specific transcription factor involved in Breg cell development remains a challenge.

**Figure 1:**
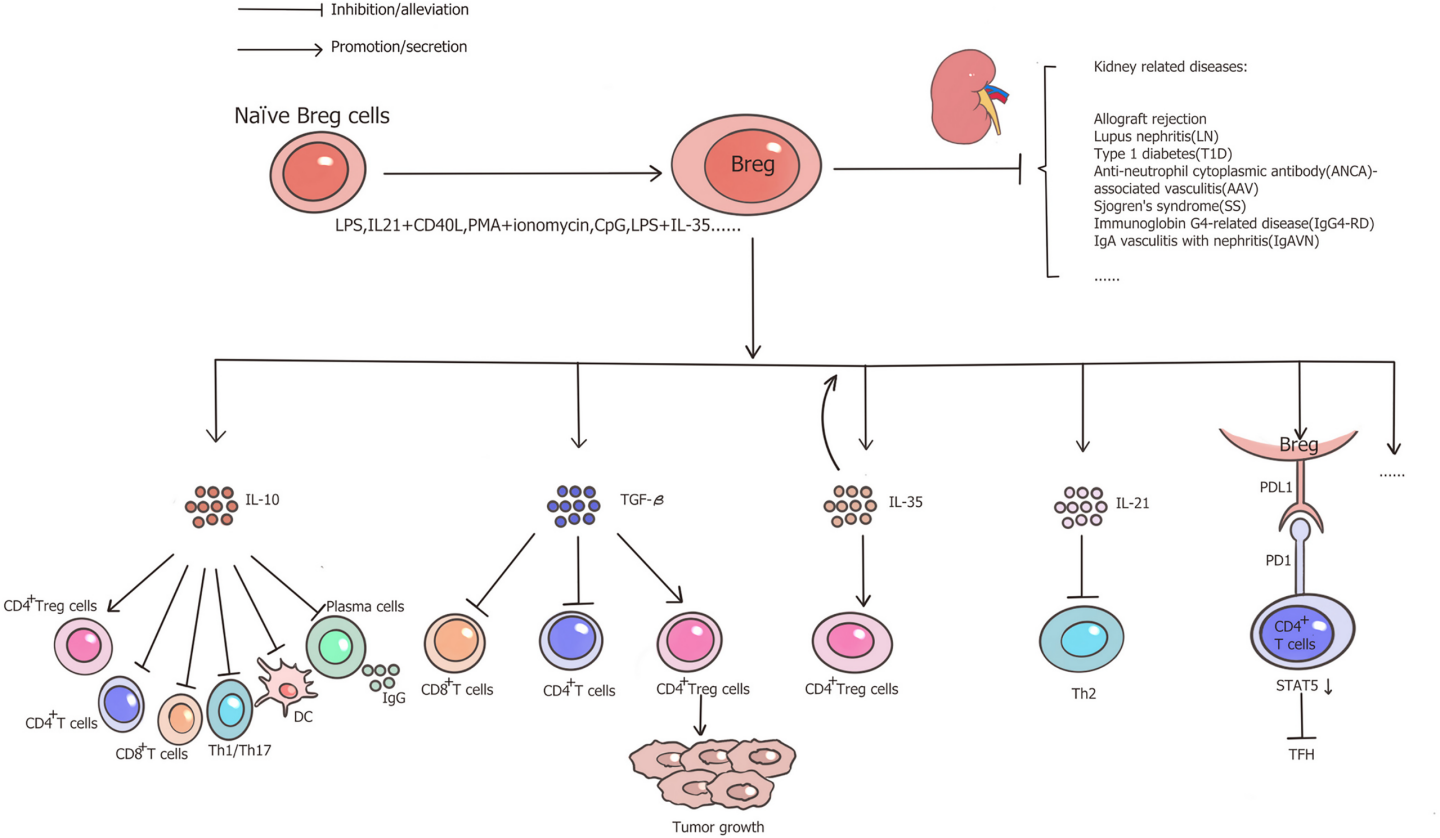
Functional properties of Breg cells and role in kidney-related diseases. Through the secretion of IL-10, IL-35, TGF-*β*, or the PD1/PDL1 pathways, Breg cells facilitate Treg cells, impair CD4^+^ T cells, CD8^+^ T cells, Th1/Th17, DC and IgG production from plasma cells, therefore, Breg cells play a protective role in allograft rejection and several kidney-related inflammatory diseases.

In summary, Breg cells are a robust inhibitory phenotype of B cells that secrete several suppressive cytokines, such as IL-10, TGF-*β*, and IL-35. Among these, IL-10-producing B cells are the most extensively studied cells in research. These cells have been proven to exhibit inhibitory functions in autoimmune diseases, inflammation, and transplantation. Its role in transplantation is a topic of immense interest, with Breg cells being comprehensively studied for their crucial role in countering graft rejection and inducing tolerance. The activation of tBreg cells has also attracted attentions of many researchers in cancer immunology field. The protective role of Breg cells have been well studied in many kidney-affecting diseases such as SLE, T1D, AAV and other inflammatory diseases, also including kidney transplantation (**Figure 1**). Cell therapy with *in vitro* induction of effective Breg cells to alleviate immune responses and to induce tolerance in clinical settings could be a major focus for future studies in kidney.

## Author Contributions

WL and HZ drafted the manuscript. ZL generated the figure. GL, WY, and LP revised the manuscript. HD designed the outline of the manuscript and revised the manuscript. All authors contributed to the article and approved the submitted version.

## Funding

This work is supported by the National Science Foundation of China (81900370, 81800664, 81970655, 82070776), Natural Science Foundation of Hunan Province of China (2019JJ50842), and Huxiang Young Talents of Hunan Province (2019RS2013).

## Conflict of Interest

The authors declare that the research was conducted in the absence of any commercial or financial relationships that could be construed as a potential conflict of interest.
